# Chilaiditi syndrome in a Nepalese girl - A potential mislead!

**DOI:** 10.1016/j.ijscr.2022.106808

**Published:** 2022-01-31

**Authors:** Aakash Mishra, Ashish Lal Shrestha

**Affiliations:** a Kathmandu Medical College Teaching Hospital, Kathmandu, Nepal; b Department of Pediatric and Neonatal Surgery, Kathmandu Medical College Teaching Hospital, Kathmandu, Nepal

**Keywords:** Chilaiditi's sign, Chilaiditi syndrome, Colonic interposition, Left-sided Chilaiditi, Pneumoperitoneum

## Abstract

**Introduction:**

Chilaiditi's Sign (CS) is a radiological finding on an abdominal radiograph due to colonic interposition between the liver and the diaphragm giving a false impression of free intra peritoneal air. When accompanied by abdominal symptoms, a syndrome with the same name is diagnosed. As elusive a finding, it can be a source of diagnostic misinterpretation causing an alarming illusion of a deceptive emergency.

**Case presentation:**

We report a four-year-old girl presenting with an acute abdomen in a background of long-standing constipation and an illusory radiograph suggesting left-sided, free sub diaphragmatic air. Further imaging with a CECT confirmed mesenteric lymphadenitis without free peritoneal air and chronic constipation as a cause of symptoms. Expectant management resulted in gradual clinical improvement.

**Discussion:**

Chilaiditi's syndrome or CS is more often described in adults and more frequently over the right side, nevertheless, can occur in children too. Knowledge of its occurrence can avoid unnecessary surgical intervention.

**Conclusion:**

While evaluating a child with acute abdomen, if the finding of a radiographic free sub diaphragmatic air is discordant with clinical signs, further imaging to rule out CS is important to avoid unnecessary exploration.

## Highlights

• Subdiaphragmatic radiolucency due to colonic interposition is termed as Chilaiditi’s sign.
• Chilaiditi’s sign, often described on the right side, can also occur on the left.
• If the clinical and radiological pictures are discordant, Chilaiditi’s syndrome should be considered.
• Conservative treatment has been proven to be effective in cases of Chilaiditi syndrome.

## 1 Introduction

Chilaiditi's sign (CS) is a rare radiological observation that was originally defined by the radiologist Demetrius Chilaiditi in 1910. CS is reported when air under the diaphragm is seen as a result of colonic interposition between the liver and diaphragm [1]. The hepatic flexure of the colon is most commonly interposed; the interposition of the splenic flexure of the colon and small bowel has been also identified infrequently [2], [3].

Radiographic evidence of colonic interposition along with clinical symptoms is referred to as Chilaiditi syndrome [1]. Chilaiditi syndrome, a cause of pseudo pneumoperitoneum is of utmost importance in clinical practice, as the differential diagnoses such as true pneumoperitoneum for gastrointestinal perforation, diaphragmatic hernia, and subphrenic abscess needs to be excluded immediately because these conditions require immediate surgical care and hover grave prognosis if not intervened early [1], [3].

We report a case of a four-years-child presenting with long-standing constipation and acute abdominal pain with a radiograph suggestive of air under the left hemidiaphragm, following the SCARE 2020 guidelines [4].

## 2 Case presentation

A four-year-old girl was evaluated for sudden onset severe periumbilical pain that later migrated towards the left lower abdomen for a day associated with multiple episodes of non-bilious and non-projectile vomiting. She did not have associated fever, abdominal distension, jaundice or urinary complaints although a history of similar pain of lesser severity and frequency was reported in the past associated with intermittent episodes of constipation that subsided after defecation.

She was hemodynamically stable except for tachycardia of 120 beats per minute. Abdominal examination revealed mild tenderness over the left entire abdomen without guarding, distension or palpable organomegaly. Bowel sounds were normal and the rest of the systemic examination was unremarkable.

Her hemogram showed a hemoglobin of 13.9 g/dl, leukocytosis with a white cell count of 12,720/cu.mm, (differential count: Neutrophils 82%/Lymphocytes 15% /Monocytes 3%) and C - reactive protein (CRP) was negative. Renal function tests and urinalysis were within normal limits.

**Fig. 1:**
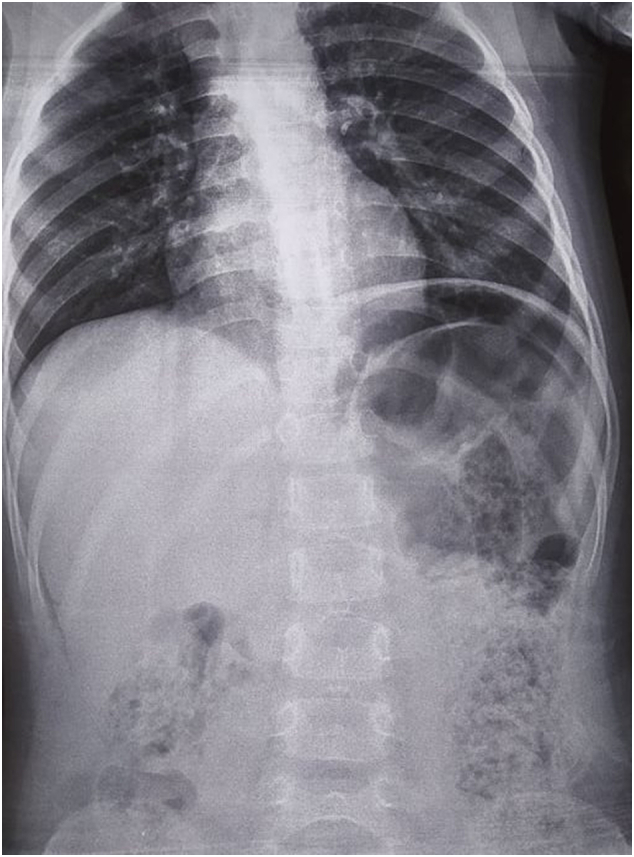
Left pseudopneumoperitoneum (Chilaiditi's sign).

A plain abdominal erect radiograph revealed a doubtful free air under the left hemi diaphragm and colonic fecal loading (Fig. 1).

Abdominal sonography suggested multiple enlarged mesenteric lymph nodes the largest measuring 17 × 14 mm in size and absence of inflamed appendix. In view of a doubtful pneumoperitoneum that was discordant with clinical findings, a contrast enhanced computed tomography (CECT) scan of the abdomen was obtained that confirmed mesenteric lymphadenitis without free peritoneal air. A final diagnosis of Chilaiditi syndrome was made with underlying mesenteric lymphadenitis and constipation.

**Fig. 2:**
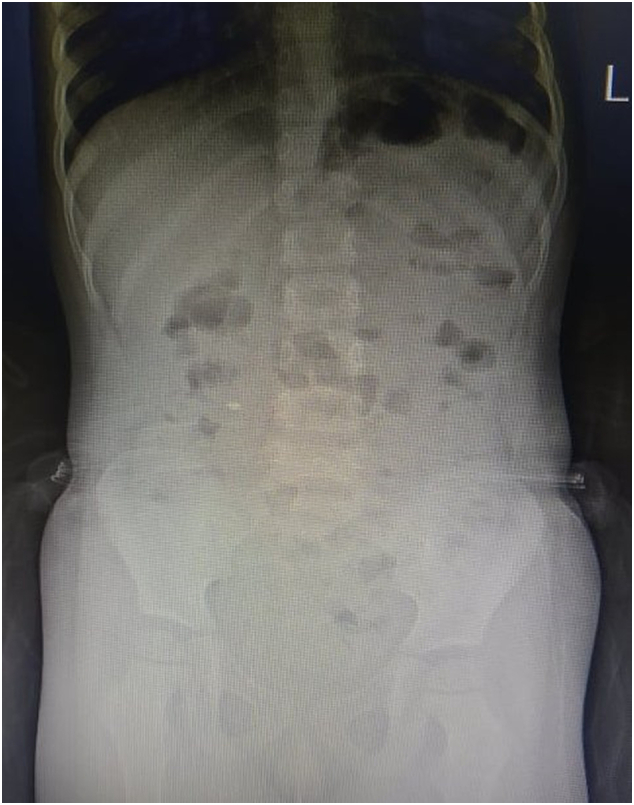
Resolution of Chilaiditi's sign at one month radiograph.

Mesenteric lymphadenitis was treated with bed rest, hydration and analgesics while stool softeners and enemas were given for constipation. With this, gradual symptomatic improvement was noted. At discharge, long term measures in the form of dietary modifications and laxatives were advised. At follow-up a week and a month later, she remained symptom-free. A repeated abdominal radiograph at one month showed a decrease in colonic fecal loading and subsequent resolution of CS (Fig. 2). The patient remains asymptomatic and disease-free for 12 months of follow-up.

## 3 Discussion

Radiological sub diaphragmatic free air is a surgical emergency and most often attributable to bowel perforation unless otherwise proved. Apart from this, other conditions like sub diaphragmatic abscess can also present with similar findings [1]. As important it is to establish the diagnosis of hollow viscous perforation, a matter of equal gravity is to avoid a negative abdominal exploration. The possibility of a false pneumoperitoneum or CS should always be thought of when abdominal signs are discordant with clinical symptoms or radiological findings [2].

CS first described by Demetrius Chilaiditi in 1910 is an uncommon radiological finding with a global incidence of 0.25–0.28% and poorly understood etiology [2]. Seemingly more common in adults, mostly men (Male:Female = 4:1) it tends to present at a median age of 60 years [2]. It is commonly described over the right side than left [2], [3]. This may represent one of the few cases of left sided CS reported globally in a child and probably the first one from Nepal.

The individuals are usually asymptomatic and the sign is often observed on the chest or abdominal radiographs incidentally. The symptomatic ones however can have a more dramatic presentation called Chilaiditi syndrome; the problems varying between mild abdominal pain, nausea, vomiting, loss of appetite, bloating, flatulence and constipation to surgical emergencies, like bowel obstruction and perforation. Interestingly, respiratory distress, chest pain and dyspnea have also been rarely described [5], [6]. Owing to its rarity, intermittency in occurrence and non-specific presentation CS can be a potential mislead [1], [2], [3]. The diagnosis is largely clinico-radiological, wherein one or more imaging modalities may be required, such abdominal radiographs, ultrasonography or computed tomography (CT) scan.

A plain radiograph is the initial investigation and reveals characteristic air below the diaphragm with visible colonic haustra that does not seem to follow a postural pattern. A cross sectional imaging like CT is mandatory to rule out perforated viscous in the background of abdominal symptoms and signs; more so, when the plain radiograph and ultrasound are inconclusive [1], [2], [3]. In children with abdominal pain, owing to the inadequacies in history and difficulties in interpreting physical signs, CT can be a useful non-invasive modality to avoid missing a sinister pathology and to confirm Chilaiditi syndrome.

Long-standing constipation as an acquired cause of Chilaiditi syndrome causing colonic elongation, redundancy, anatomic distortion and pathological interposition resulting in CS has been implicated [1], [3], [7]. The same was observed with our patient who had been having intermittent episodes of functional constipation since one year of age.

Chilaiditi syndrome may infrequently need surgical rectification in the form of colopexy or resection when conservative measures fail due to associated volvulus, intestinal ischemia or perforation [3], [8]. However, with our patient, following CT confirmation, an expectant form of management was employed that resulted in gradual improvement.

Also considering constipation as the cause of CS, long-term management involving dietary modifications and laxatives were advised. Resolution of CS and reduction in colonic fecal loading in the subsequent abdominal radiographs confirmed objective improvement with long-term benefits.

## 4 Conclusion

When evaluating a child with abdominal pain and long-standing constipation, if radiological free air is discordant with clinical findings, the possibility of Chilaiditi Syndrome should be suspected. Further confirmation with CT and conservative care can often avoid unnecessary exploration.

## Funding

None.

## Ethical approval

Not required.

## Consent for publication

Written informed consent was obtained from the patient for publication of this case report and accompanying images. A copy of the written consent is available for review by the Editor-in-Chief of this journal on request.

## Authors' contribution

AM drafted the manuscript. ALS was the treating physician, senior author and supervisor and revised the manuscript.

## Registration of research studies

Not applicable.

## Note

No patient and author details are included in the figure.

## Guarantor

Aakash Mishra accepts full responsibility for the work and/or the conduct of the study, had access to the data, and controls the decision to publish.

## Provenance and peer review

Not commissioned, externally peer-reviewed.

## Declaration of competing interest

None of the authors has any conflict of interest to disclose. We confirm that we have read the Journal's position on issues involved in ethical publication and affirm that this report is consistent with those guidelines.

## References

[1] Moaven O., Hodin R.A. (2012). Chilaiditi syndrome: a rare entity with important differential diagnoses. Gastroenterol. Hepatol..

[2] Kapania E.M., Link C., Eberhardt J.M. (2018). Chilaiditi syndrome: a case report highlighting the intermittent nature of the disease. Case Rep. Med..

[3] Weng W.-H., Liu D.-R., Feng C.-C., Que R.-S. (2014). Colonic interposition between the liver and left diaphragm - management of chilaiditi syndrome: a case report and literature review. Oncol. Lett..

[4] Agha R.A., Franchi T., Sohrabi C., Mathew G., Kerwan A., Thoma A., Beamish A.J., Noureldin A., Rao A., Vasudevan B., Challacombe B., Perakath B., Kirshtein B., Ekser B., Pramesh C.S., Laskin D.M., Machado-Aranda D., Miguel D., Pagano D., Millham F.H., Roy G., Kadioglu H., Nixon I.J., Mukherjee I., McCaul J.A., Chi-Yong Ngu J., Albrecht J., Rivas J.G., Raveendran K., Derbyshire L., Ather M.H., Thorat M.A., Valmasoni M., Bashashati M., Chalkoo M., Teo N.Z., Raison N., Muensterer O.J., Bradley P.J., Goel P., Pai P.S., Afifi R.Y., Rosin R.D., Coppola R., Klappenbach R., Wynn R., De Wilde R.L., Surani S., Giordano S., Massarut S., Raja S.G., Basu S., Enam S.A., Manning T.G., Cross T., Karanth V.K.L., Kasivisvanathan V., Mei Z., The S.C.A.R.E. (2020). Guideline: updating consensus surgical CAse REport (SCARE) guidelines. Int. J. Surg..

[5] Caicedo L., Wasuwanich P., Rivera A., Lopez M.S., Karnsakul W. (2021). Chilaiditi syndrome in pediatric patients - symptomatic hepatodiaphragmatic interposition of colon: a case report and review of literature. WorldJ. Clin. Pediatr..

[6] Xu Y., Wang Q., Meng G., Li D., Ma Z., Gao P., Zhang J., Zhang Q., Su Z. (2020). A rare cause of sudden chest pain and dyspnea: a CARE-compliant case report of chilaiditi syndrome. Medicine (Baltimore).

[7] Toros A.B., Arisoy K. (2015). Solda chilaiditi: Nadir bir vaka sunumu.

[8] Blevins W.A., Cafasso D.E., Fernandez M., Edwards M.J. (2011). Minimally invasive colopexy for pediatric chilaiditi syndrome. J. Pediatr. Surg..

